# Antifungal‐Based Conservative Management of Delayed‐Onset Fungal Bronchopleural Fistula After Lung Cancer Surgery

**DOI:** 10.1002/rcr2.70635

**Published:** 2026-07-02

**Authors:** Jonggeun Lee, Ho Seong Cho, Jeong Su Cho

**Affiliations:** ^1^ Department of Thoracic & Cardiovascular Surgery, School of Medicine Pusan National University, Biomedical Research Institute, Pusan National University Hospital Busan Korea

**Keywords:** *Aspergillus fumigatus*, bronchopleural fistula, conservative management

## Abstract

Bronchopleural fistula (BPF) is a rare but potentially life‐threatening complication after pulmonary resection. When associated with Aspergillus infection, management becomes particularly challenging because of tissue necrosis and impaired bronchial healing. Although surgical intervention is generally recommended, optimal management of delayed‐onset fungal BPF remains controversial. We report two cases of postoperative *Aspergillus fumigatus* –associated BPF following lung cancer surgery that were managed primarily with antifungal therapy and pleural drainage. In the first case, a delayed‐onset BPF developed 14 months after bilobectomy and resolved completely with percutaneous drainage and prolonged voriconazole therapy without additional surgery. In the second case, limited surgical closure failed to eliminate persistent air leakage; however, subsequent antifungal therapy combined with continued drainage resulted in complete fistula closure. These cases suggest that in carefully selected patients with localized, delayed‐onset fungal BPF and clinical stability, antifungal‐based conservative management may be considered in carefully selected patients as a potential adjunct or alternative to aggressive surgical intervention. Careful patient selection and multidisciplinary evaluation are essential.

## Graphical abstract

We report two cases of delayed‐onset *Aspergillus fumigatus* –associated bronchopleural fistula following lung cancer surgery that provide insight into patient selection for conservative management.

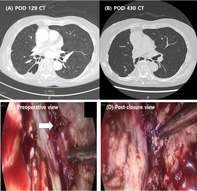

## 1 Introduction

Bronchopleural fistula (BPF) is an uncommon yet potentially life‐threatening complication following pulmonary resection, with a reported incidence ranging from 0.5% to 5% depending on the extent of surgery and patient‐related risk factors [1, 2]. Although bacterial infection represents the most frequent aetiology, fungal BPF—most commonly caused by Aspergillus species—poses unique therapeutic challenges. Fungal infection is associated with tissue necrosis, bronchial wall destruction and impaired healing of bronchial stumps or anastomoses, often resulting in persistent air leakage and chronic empyema [3, 4]. Mortality rates exceeding 30% have been reported, and most published cases require aggressive surgical interventions such as open‐window thoracostomy, muscle flap transposition, or repeated thoracotomy [1, 2].

The fundamental principles of BPF management include eradication of infection, adequate pleural drainage and definitive closure of the fistula when technically feasible [1, 2]. However, surgical intervention in patients with fungal BPF is frequently complicated by poor tissue quality, extensive adhesions and significant operative risk, particularly in delayed or post‐adjuvant settings [2]. Recently, several reports have suggested that antifungal therapy combined with effective pleural drainage may stabilize infection and, in selected cases, allow spontaneous closure of small and localized fistulas [3, 4].

Delayed‐onset BPF is generally defined as a fistula occurring more than 30 days after pulmonary resection and is often associated with chronic infection, fibrosis, or adjuvant therapy rather than early technical failure [5].

We report two cases of delayed‐onset *Aspergillus fumigatus* –associated BPF following lung cancer surgery that provide insight into patient selection for conservative management.

## 2 Case Reports

### 2.1 Case 1

**FIGURE 1:**
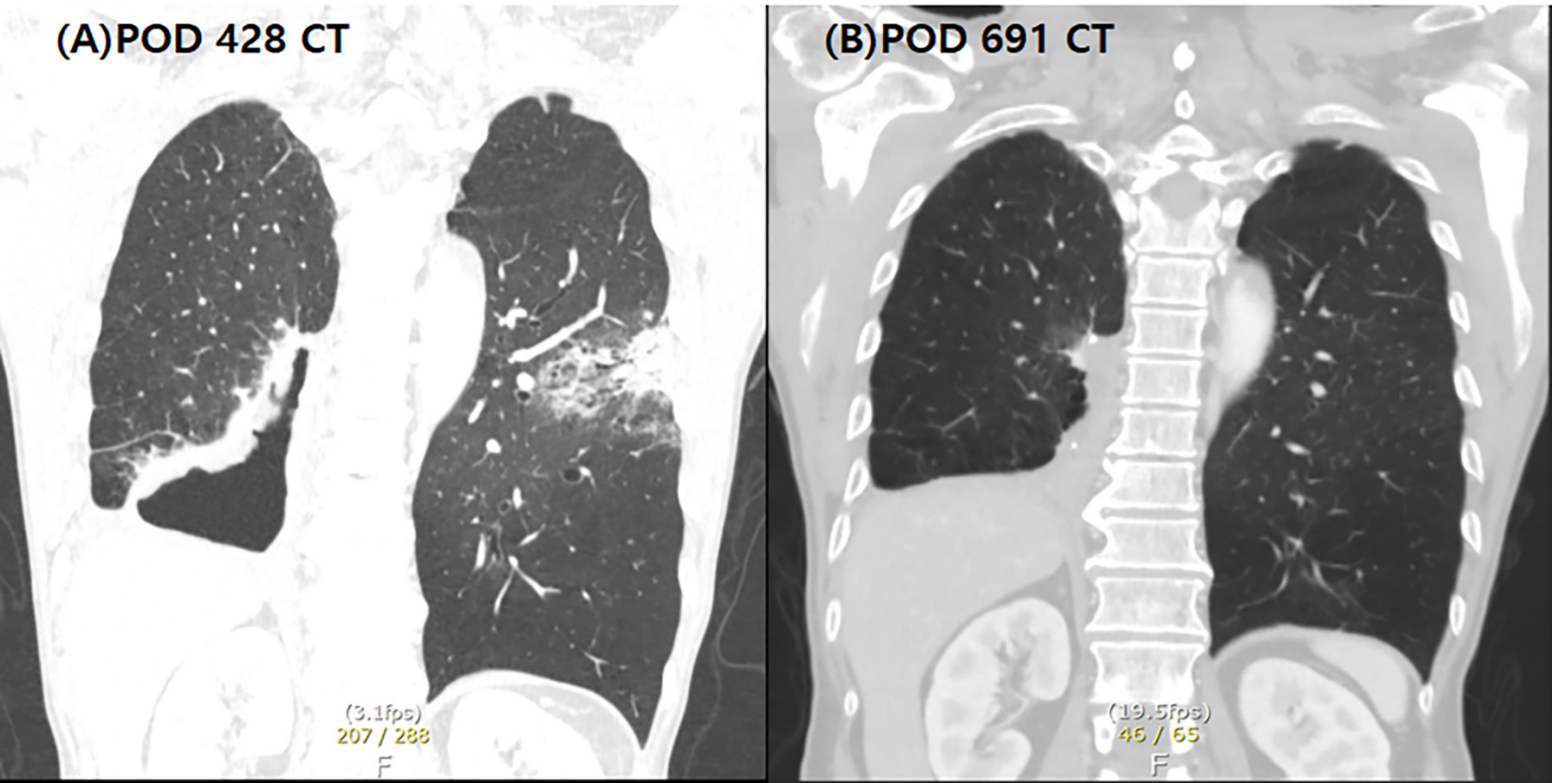
Case 1: Serial chest CT scans showing the course of the right lower hemithoracic cavity caused by *Aspergillus fumigatus* ‐associated bronchopleural fistula. (A) On postoperative day (POD) 428, CT revealed a cavitary lesion in the right lower hemithorax consistent with a bronchopleural fistula. (B) On POD 691, following long‐term voriconazole therapy, the cavity had markedly decreased and the air–fluid level resolved, allowing removal of the percutaneous catheter drain.

A 62‐year‐old man with squamous cell carcinoma of the right lung underwent video‐assisted thoracoscopic wedge bronchoplastic bilobectomy. The postoperative course was uneventful. Fourteen months later, he presented with persistent cough and pleuritic chest pain refractory to antibiotics. Chest CT revealed a cavitary lesion in the right lower hemithorax consistent with bronchopleural fistula (Figure 1A). The lesion demonstrated a small‐calibre air‐filled tract communicating between the bronchial stump and the pleural cavity, without evidence of extensive pleural contamination. Pleural cultures grew *A. fumigatus* . Conservative management was selected due to delayed onset, absence of systemic sepsis and a localized cavity amenable to drainage [6].

Percutaneous catheter drainage was instituted, and systemic voriconazole therapy was initiated. Serial imaging demonstrated gradual reduction of the pleural cavity, and air leakage resolved without further surgical intervention. Complete fistula closure was confirmed radiologically (Figure 1B) and no recurrence was observed during follow‐up.

Diagnosis was established based on pleural fluid culture. Additional fungal biomarkers such as serum galactomannan or beta‐d‐glucan were not routinely performed.

### 2.2 Case 2

**FIGURE 2:**
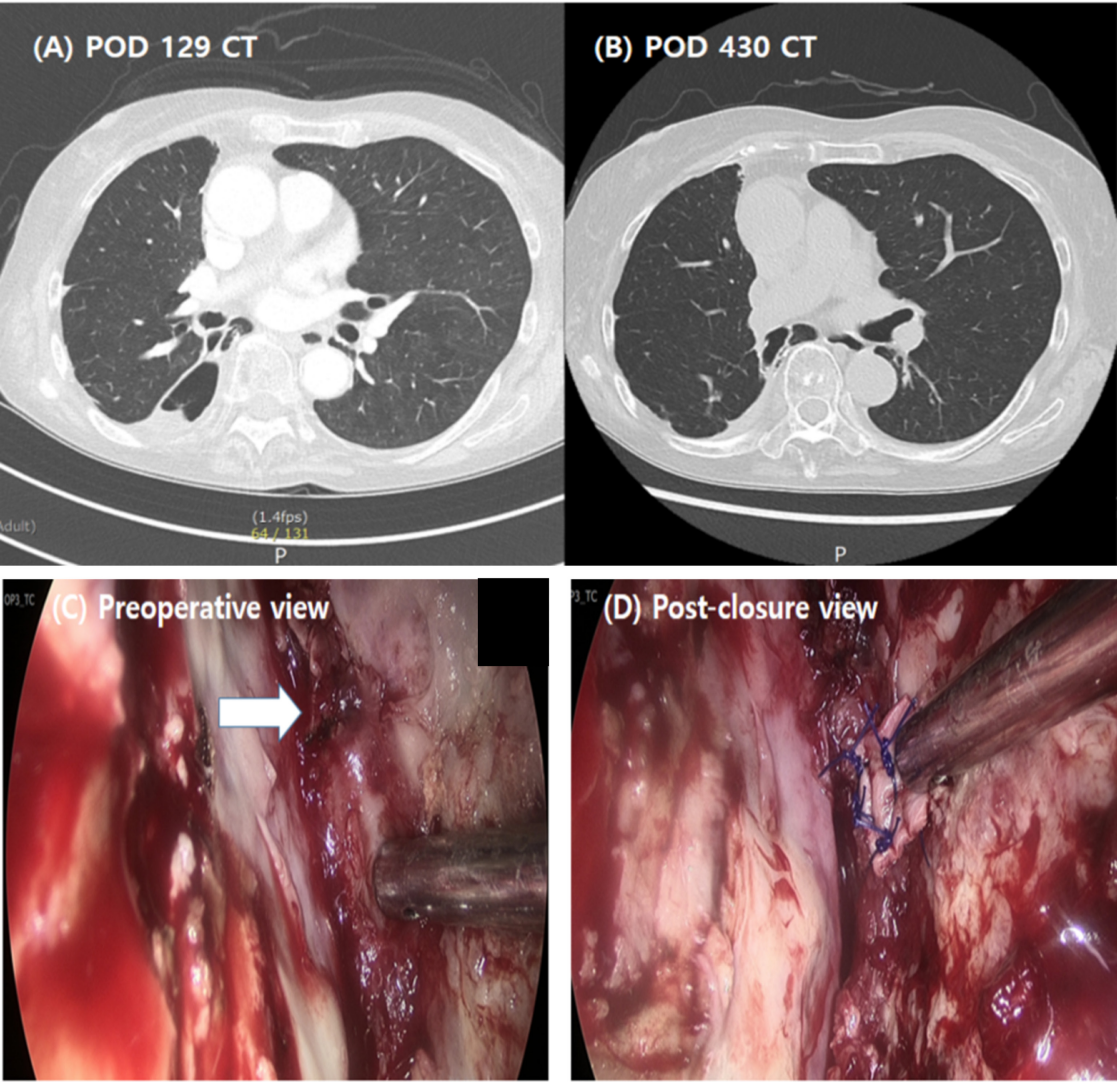
Case 2: Radiologic and operative findings in a small bronchopleural fistula with *Aspergillus fumigatus* infection following right lower lobectomy. (A) POD 129 CT shows a tiny bronchopleural fistula adjacent to the bronchial stump. (B) POD 430 CT demonstrates complete resolution after limited surgical closure and antifungal therapy. Follow‐up CT showing resolution of the cavity. (C) Preoperative operative photograph showing the fistula opening. White arrow indicates a focal defect within the debris‐filled cavity consistent with bronchopleural fistula. (D) Final operative photograph confirming complete closure.

A 74‐year‐old woman underwent right lower lobectomy for lung adenocarcinoma. Several months later, Chest CT demonstrated a small, focal air‐containing defect adjacent to the bronchial stump, consistent with a bronchopleural fistula (Figure 2A). The fistula appeared to be localized and small in calibre, without an associated large empyema cavity. Pleural cultures confirmed *A. fumigatus* . Initial drainage was performed; however, persistent air leakage prompted limited surgical closure (Figure 2C,D). The surgical approach was performed via thoracoscopic re‐entry. Intraoperatively, dense adhesions and inflamed fibrotic tissue were noted around the bronchial stump. A small fistulous opening, approximately 2 mm in size, was identified adjacent to the stump, without evidence of extensive pleural contamination. The defect was closed primarily using interrupted sutures with surrounding tissue reinforcement.

Despite surgical repair, air leakage recurred shortly thereafter, suggesting inadequate mechanical closure. Voriconazole therapy was subsequently initiated and combined with continued pleural drainage. The air leak gradually resolved, and follow‐up imaging confirmed complete closure of the fistula (Figure 2B).

Diagnosis was established based on pleural fluid culture. Additional fungal biomarkers such as serum galactomannan or beta‐d‐glucan were not routinely performed.

## 3 Discussion

These cases suggest that delayed‐onset fungal BPF may represent a biologically distinct entity compared with early postoperative BPF. Early fungal BPF is typically associated with friable tissue and acute empyema, often necessitating aggressive surgical intervention. In contrast, delayed‐onset fistulas occurring months after resection may arise within a fibrotic postoperative environment characterized by mature adhesions and relatively small, stable defects [3, 4]. This distinction may partly explain the favourable response to conservative management observed in our patients. Based on our cases and previous reports, conservative management may be considered in carefully selected patients with the following features: (1) delayed onset (> 30 days after surgery), (2) small‐calibre and localized fistula, (3) absence of systemic sepsis and (4) adequate pleural drainage with controlled infection.

In Case 1, antifungal therapy and adequate drainage were sufficient to achieve spontaneous closure. In Case 2, limited surgical repair alone did not provide durable resolution, and infection control through antifungal therapy appeared decisive. The failure of the initial surgical repair in Case 2 may be attributed to ongoing fungal infection and inflamed, friable tissue around the bronchial stump, which likely impaired tissue healing and suture integrity, resulting in persistent air leakage despite mechanical closure.

These observations highlight that mechanical closure without adequate control of fungal infection may be insufficient and that infection control should precede or accompany surgical closure, particularly in fungal BPF [4].

Previous reports have emphasized aggressive surgical management for fungal BPF due to high mortality; however, successful conservative management has been reported in selected cases with localized disease and controlled infection [2]. Standard surgical approaches for BPF include open‐window thoracostomy, muscle flap transposition and endobronchial interventions. While these strategies aim to achieve definitive closure and infection control, they are often associated with significant morbidity, prolonged hospitalization and reduced quality of life. In contrast, conservative management may offer a less invasive alternative in carefully selected patients with localized disease and stable clinical conditions. However, it should not replace surgical intervention in patients with large fistulas, uncontrolled sepsis, or clinical instability.

Although fungal biomarkers such as serum galactomannan and beta‐D‐glucan may aid in the diagnosis of invasive fungal infections, their diagnostic value in localized pleural aspergillosis remains limited, with pleural fluid culture remaining the primary diagnostic modality [3]. In our cases, diagnosis was primarily based on pleural fluid culture and clinical context rather than serologic markers.

Voriconazole remains the first‐line antifungal agent for invasive *Aspergillus* infection, demonstrating superior efficacy and survival compared with amphotericin B [7]. Current guidelines recommend therapeutic drug monitoring to maintain serum trough concentrations between 1.0 and 5.5 μg/mL to optimize efficacy while minimizing toxicity [7, 8]. Prolonged therapy combined with close radiologic surveillance is critical to prevent recurrence.

A practical management approach may include: (1) assessment of clinical stability, (2) ensuring adequate pleural drainage, (3) initiation of systemic antifungal therapy and (4) consideration of surgical intervention in cases of large fistula, uncontrolled sepsis, or failure of conservative management [2].

This study has several limitations. First, it is based on only two cases, which limits the generalizability of our findings. Second, the applicability of conservative management may be restricted to carefully selected patients with specific clinical characteristics. Further studies with larger cohorts are needed to validate these observations.

In conclusion, delayed‐onset fungal BPF following lung cancer surgery may be amenable to antifungal‐based conservative management in carefully selected clinical settings. Adequate drainage, prolonged antifungal therapy and multidisciplinary evaluation are essential to achieving durable fistula closure while minimizing unnecessary surgical risk.

## Author Contributions

**Jonggeun Lee:** writing – original draft, writing – review and editing. **Ho Seong Cho:** supervision, writing – original draft. **Jeong Su Cho:** investigation, resources, supervision. All authors read and approved the final version of the manuscript.

## Funding

The authors have nothing to report.

## Ethics Statement

Review by the institutional review board was waived due to the retrospective nature of this case report. Written informed consent was obtained from the patients.

## Consent

The authors declare that written informed consent was obtained for the publication of this manuscript and accompanying images using the form provided by the Journal.

## Conflicts of Interest

The authors declare no conflicts of interest.

## Data Availability

Data sharing not applicable to this article as no datasets were generated or analysed during the current study.
